# The Medical Association of Georgia

**Published:** 1887-03

**Authors:** 


### THE MEDICAL ASSOCIATION OF GEORGIA.

The forthcoming meeting of the Medical Association of Georgia, on April 20 in the city of Atlanta, presents some points for the consideration of the entire medical profession of this State, and it is hoped that, not only those already enrolled on the list of members will attend, but that many who have not heretofore joined the Association may avail themselves of the occasion to co-operate in the work of medical progress.

While it is desirable that each branch of medical science shall be represented by papers from those whose experience fits them for preparing elaborate articles, it is to be expected that a large proportion of our practitioners may contribute reports of cases which shall prove instructive ; and, though the time is short, we would urge upon our brethren throughout the State to come forward and help in sustaining the character of this State organization.

It is in contemplation also to effect a radical reform in the relation of local societies to the Association, and to perfect the plan of organization upon the basis of the Alabama Medical Association. The committee entrusted with this matter at the last annual meeting is expected to present a report embodying the modifications that may be considered expedient, and it is especially desirable that every portion of the State shall have representatives present to deliberate upon these changes. Physicians from every town and county neighborhood ought to be in attendance at this meeting with a view to give expression to their convictions as to the proposed reorganization, and we trust that a large proportion of the representative men from the several sections of the State may attend the meeting and lend their counsels in the discussion touching the expediency and practicability of the Alabama plan of organization. Don't let any one stay away because of the impression that he may not have an influential position among his fellows, as in the medical ranks all are equal, and the voice of everyone should be heard in matters of this kind, which look to the relations of the several parts to the whole body. The efficiency of our medical organization must depend largely upon the hearty support of the members, and if a larger number of the physicians of the State join the Association better results can be secured. While the material for successful operation is abundant in Georgia, there has been such a lukewarmness on the part of many physicians, especially in country places, as to retard this co-operative effort to build up an organization calculated to extend our usefulness in the State and to enhance the standing of the profession in other States, we are in danger of being left behind in the competition of State organizations, if something is not done now towards bringing Georgia to the front ; and hence the vital importance of rallying in force at the approaching meeting in Atlanta.

There are considerations in respect to the International Medical Congress, which meets in September at Washington City, which appeal to all physicians throughout this country to declare their allegiance anew to the great principles of equal rights among medical men in upholding the organization which has been completed for carrying on the work of that body. With a view to secure the best results in the scientific and practical outcome of the Congress, the official positions of the various sections have been filled by efficient representatives of the profession at home and abroad. so that no doubt exists as to the recognition of this Congress by the medical world. A large number of the most prominent members of the medical profession in foreign lands have signified their intention to be present and participate in the proceedings of the Congress, and it is confidently expected that many of those amongst us, who, from various causes, have thought proper to stand aloof from any official connection with the organization, may conclude to contribute to the scientific work of the Congress at Washington.

Under all the surroundings of the past and present aspects of this International undertaking, it would have a good effect upon the outside world if the State Medical Association should endorse in due form the present organization, and doubtless the voice of the Medical Association of Georgia will be given in support of the existing order of things so as to uphold and strenghten the arms of those who have been struggling for the honor and dignity of all classes of regular physicians in this country. A small faction of malcontents should not, and must not, prevent the hearty co-operation of the great mass of the profession in securing the complete success of the Ninth International Medical Congress.

